# Scaling-Laws of Flow Entropy with Topological Metrics of Water Distribution Networks

**DOI:** 10.3390/e20020095

**Published:** 2018-01-30

**Authors:** Giovanni Francesco Santonastaso, Armando Di Nardo, Michele Di Natale, Carlo Giudicianni, Roberto Greco

**Affiliations:** 1 Dipartimento di Ingegneria Civile, Design, Edilizia e Ambiente, Università degli Studi della Campania “Luigi Vanvitelli”, via Roma 29, 81031 Aversa, Italy; 2 Action Group CTRL+SWAN of the European Innovation Partnership on Water, EU, B-1049 Brussels, Belgium

**Keywords:** scaling laws, power laws, water distribution networks, robustness, flow entropy

## Abstract

Robustness of water distribution networks is related to their connectivity and topological structure, which also affect their reliability. Flow entropy, based on Shannon’s informational entropy, has been proposed as a measure of network redundancy and adopted as a proxy of reliability in optimal network design procedures. In this paper, the scaling properties of flow entropy of water distribution networks with their size and other topological metrics are studied. To such aim, flow entropy, maximum flow entropy, link density and average path length have been evaluated for a set of 22 networks, both real and synthetic, with different size and topology. The obtained results led to identify suitable scaling laws of flow entropy and maximum flow entropy with water distribution network size, in the form of power–laws. The obtained relationships allow comparing the flow entropy of water distribution networks with different size, and provide an easy tool to define the maximum achievable entropy of a specific water distribution network. An example of application of the obtained relationships to the design of a water distribution network is provided, showing how, with a constrained multi-objective optimization procedure, a tradeoff between network cost and robustness is easily identified.

## 1. Introduction

The topology of water distribution networks (WDN) is being deeply studied with respect to its relationship with their robustness, i.e., their capability of effectively delivering the demanded flows to the users with the required pressure under unfavorable operating conditions [1]. In fact, evaluating the performance of a WDN requires the complex calibration of a hydraulic model of the network, and often a number of time-consuming simulations. Hence, establishing relationships, linking topological metrics of a WDN, easily achievable from the mere knowledge of the network layout, with its hydraulic behavior, would represent a powerful tool for the design, rehabilitation and management of WDN. In this respect, aiming at quantitative comparison of different network layouts, it is important to understand how topological metrics change with the size of the considered network.

In fact, the size variation of a system can cause changes in the order of predominance of physical phenomena; this is called scaling effect, and the laws that govern such an effect are called scaling laws. The scaling laws are relationships linking any parameter associated with an object (or system) with its length scale [2]. They constitute a very useful tool to predict the behavior and the properties of a large system by experimenting on a small-sized scale model, since the characteristics of a system can be expressed through various parameters in such a way that any change in size (i.e., scale) does not affect the magnitudes of these quantities. Scaling laws represent useful tools for understanding the interplay among various physical phenomena and geometric characteristics of complex systems, and often it happens that simple scaling laws can provide clues to some fundamental aspects of the system. In many fields, scaling laws have been identified. For example, scaling laws have been experimentally determined over a huge range of scales in probability distributions describing river basin morphology [3], whose geometrical description is of great importance for a deeper understanding of how some related natural events occur. The existence of a scaling law relating point precipitation depth records to duration has been known for at least 60 years through published tabulations of data and the associated graphs [4,5], even if there is no explanation of the mechanism underlying this remarkably robust relationship, making it even more tantalizing [6]. Scaling laws have been also identified in fluid mechanics, to describe turbulent energy distribution across scales [7,8], and in meteorology, to describe scaling of clouds [9], atmospheric variability [10], and fluctuations of Arctic sea ice [11]. In the field of network topology, it has been found that many real networks exhibit power–law shaped node degree distribution, where the degree is the number of connected links to each node. Such networks have been named scale-free networks [12], because power–laws have the property of retaining the same functional form at all scales. These networks result in the simultaneous presence of a few nodes (the hubs) linked to many other nodes, and a large number of poorly connected elements [13]. The World Wide Web (WWW) is one of the most famous scale-free networks. It is formed by the hyperlinks between different Web pages, and, with more than 10^8^ nodes, it is the largest network ever studied.

Differently, water distribution networks (WDN) do not present hubs, as each node is connected only to a few nodes located in its immediate surroundings. The connections between nodes in a WDN ensure multiple possible flow paths, so to cope with abnormal working conditions, such as unexpected water requests by the users and failure of some elements [14]. In this respect, several topological metrics aimed at quantifying WDN connectivity have been proposed as proxies for network robustness and reliability [15].

Reliability, in a WDN, can be defined as the probability of the system being capable of supplying the water demands both under normal and abnormal conditions [16,17]. The assessment of reliability is influenced by many factors: spatial and temporal demand distribution, possible failure of one or more components, pressure-flow relationship, connectivity of the network, etc. Therefore, there is not an established measure of WDN reliability, and a review of different methods to evaluate it can be found in [18]. Reliability measures are categorized into three groups: topological, hydraulic and entropic. Topological reliability is based on the probability of node connectivity/reachability [19]; hydraulic reliability is focused on the probability of delivering design water demands, (e.g., [17]); and the last category adopts the informational entropy as a surrogate of the reliability [18]. 

The concept of informational entropy [20] has been widely applied in hydraulics and hydrology (i.e., to estimate velocity distribution in open channels, suspended sediment concentration profile, suspended sediment discharge, or precipitation variability, moisture profiles, etc.) [21]. In the field of WDN, Shannon’s entropy has been proposed as a measure of connectivity and so as a proxy for reliability [22].

The adoption of entropy as a surrogate for network reliability was investigated by several authors [23,24,25,26]. The basic idea is that entropy is a measure of the uniformity of pipe flow rate [27], thus it is related to looped network redundancy, which makes it potentially more capable of facing unfavorable working conditions, such as concentrated peaks of demand or failure of pipes (e.g., [1]). Hence, redundancy increases network robustness, and so, indirectly, its reliability.

Hence, many studies [28,29,30] have proposed multi-objective optimization for water distribution network design or rehabilitation based on minimizing costs for construction, operation, and maintenance, coupled with the maximization of the entropy as a measure of robustness.

Traditionally, the robustness of water distribution networks was assured by means of densely looped layouts, so to provide alternative paths for each demand node [31]. More recently, Di Nardo et al. [32] have studied the topological redundancy of a water supply network, with regard to pipe failures, applying the complex network theory [33,34]. In fact, many water supply systems consisting of up to tens of thousands nodes and hundreds of looped paths can be considered as complex networks [13]. Thus, it is possible to compute topological metrics [32,35,36,37] to analyze the robustness of a water distribution network. 

Recently, comparisons between entropy and other indirect measures of robustness [1,26,38,39,40] such as resilience index [41], network resilience [42] and Surplus Power Factor [43] have been proposed, but the obtained results are contradictory. According to some authors [26,38,39], informational entropy is a good measure of network robustness. Conversely, other studies indicate that the resilience index estimates better the network hydraulic performance than entropy in the case of pipe failures [1] and for multi-objective design optimization [40].

The advantage of using informational entropy to evaluate network robustness is that only pipe flows and topology are required for its computation [39], while the main drawback is that there is not a reference value of entropy allowing for defining an acceptable level of robustness for a given WDN, nor to compare different WDN layouts. In this respect, the definition of scaling laws of flow entropy with the topological dimension of the network could be useful for WDN design and rehabilitation purposes.

This work investigates the possible relationship between topological metrics, borrowed from complex network theory, and flow entropy, through the analysis of the values that they assume for several WDNs, both real and synthetic. In particular, for each network, five of the coarsest topological characteristics of a network, the number of nodes *n* and links *m*, the average node degree *k*, the link density *q* and the average path length *APL* have been calculated. The results show that the flow entropy of a WDN is strongly linked to its size and topology, and that it can be expressed as a function of topological metrics. Furthermore, the maximum achievable flow entropy value has been calculated for each WDN. Scaling-laws of flow entropy with the size of the networks have been identified. Two examples of the application of the obtained results to the design of WDNs are finally provided.

## 2. Methods 

The study of WDN using innovative topological metrics, borrowed from the theory of complex networks [13], already led to interesting results for the analysis of water network vulnerability [32,35,44], as well as for water network partitioning [45,46]. In the following sections, the topological and entropy metrics used in this paper are briefly described, and finally the deviation of actual entropy from maximum entropy is introduced as a possible measure of network robustness.

### 2.1. Topological Metrics

The average node degree, *k*, represents the mean number of links concurring in the nodes of the network, and is given by:

(1) $$k=\frac{2\cdot m}{n}$$

in which *n* is the number of nodes and *m* the number of links of the network.

The link density, *q*, expresses the ratio between the total number of network edges and the number of edges of a globally coupled network with the same number of nodes, thus providinga measure of network redundancy:

(2) $$q=\frac{2\cdot m}{n\cdot \left(n-1\right)}$$

The average path length, *APL* [33], is the average number of steps along the shortest paths between all possible pairs of nodes in the network:

(3) $$APL=\frac{\sum_{\forall s\neq t}\sigma \left(s,t\right)}{\frac{1}{2}n\cdot \left(n-1\right)}$$

where *σ*(*s*,*t*) is the number of edges along the shortest path connecting node *s* to node *t* (when there is no path between a pair of nodes, the path length is assumed to be infinite) [47]. A short average path length indicates a more interconnected network, while a long one indicates greater overall topological distances between nodes. Consequently, a network with a large *APL* value may be considered more fragmented [48].

### 2.2. Entropic Metrics

The Shannon’s information entropy [49] is a statistical measure of the amount of uncertainty associated with the probability distribution of any discrete random variable, defined as follows:

(4) $$E=-\overset{l}{\underset{k=1}{\sum }}p_{k}\ln p_{k},$$

where *E* is the entropy, *p_k_* is the probability, and *l* is the number of values that the variable can assume. Tanyimboh and Templeman [28], with the use of the conditional entropy formula of [50], considered all the possible flow paths from sources to demand nodes, and introduced the flow entropy S of a water distribution system by defining the probability of the water to flow along the *k*-th path as the ratio between the flow rate reaching the end node of the path and the total delivered flow rate [42]. The following recursive formula [24] allows the calculation of *S*, which is regarded as a measure of pipe flow rates uniformity:

(5) $$S=-\overset{NS}{\underset{i=1}{\sum }}\frac{Q_{i}}{T}\ln\left(\frac{Q_{i}}{T}\right)-\frac{1}{T}\overset{NN}{\underset{j=1}{\sum }}T_{j}\left[\frac{Q_{j}}{T_{j}}\ln\left(\frac{Q_{j}}{T_{j}}\right)+\underset{ji\in N_{j}}{\sum }\frac{q_{ji}}{T_{j}}\ln\left(\frac{q_{ji}}{T_{j}}\right)\right]$$

On the right hand side of Equation (5), the first term is the entropy of supply nodes and the second is the entropy of demand nodes; *NS* is the number of supply nodes; *T* is the total supplied flow rate; *NN* is the number of demand nodes; *Q_i_* represents the inflow at the *i*-th source node; *T_j_* is the total flow rate reaching the *j*-th demand node; *Q_j_* is the water demand at the *j*-th demand node; *q_ij_* is the flow rate in the pipe connecting node *j* with surrounding node *i*; and *N_j_* is the number of pipes carrying water from the *j*-th demand node towards other surrounding nodes.

The data required to assess the flow entropy are the topological layout, the water supply and the demand at all nodes, and the flow direction along each pipe. To this purpose, the hydraulic simulation of the network, carried out with the solver EPANET 2 [51], provides the flow rate and direction along each pipe.

### 2.3. Maximum Entropy and Network Robustness

The maximization of Equation (3) can be used to compute the maximum value of the flow entropy, *MS*, and in this case only the source flow rates, the water demands at nodes and the flow directions along the links are required. Specifically, *MS* is here computed here by means of a non-iterative procedure for multi-source networks, proposed in [52]. The entropy deficit, i.e., the deviation between the flow entropy *S* and the corresponding values of *MS*, given by Equation (5), is assumed to be representative of how much a network is robust, based on the idea that networks, designed to supply maximum entropy flows, would be the most robust for a given source pressure excess compared to the design pressure at nodes [23].

(6) $$∆S=1-\frac{S}{MS}$$

## 3. Results and Discussions

Topological metrics and flow entropy metrics were computed for a set of 22 WDNs, both real and synthetic. The maximum entropy *MS* of each network was calculated adopting the same flow directions along the pipes as for the calculation of flow entropy *S* (i.e., the directions provided by the hydraulic simulation of the network for the actual set of pipe sizes). Therefore, the obtained *MS* cannot be considered as the maximum possible values of flow entropy, as a different choice of flow directions could lead to a higher value of *MS*. However, as the flow directions are mainly dictated by the position of sources and demand nodes, and by the assumed water demand at nodes, it is expected that flow directions would be only slightly (and locally) affected by changes in the size of some of the pipes. In Table 1, the computed values of the metrics are reported for all the considered networks.

The set of networks used as case study includes water distribution networks with very different characteristics, as indicated by the very different values assumed by the metrics:

- dimension: the smallest network has a number of nodes *n* = 6 (Two Loop), while the largest has *n* = 1890 (Exnet);
- layout: looped networks as well as branched ones are included, i.e., Balerma Irrigation can be considered a tree-network, while networks such as Parete and Sector Centro Real are very looped; compact and elongated networks are included, with low values of APL coupled with high values of density being representative of compact network layouts;
- robustness: the set of networks includes systems with very small deviation of actual entropyfrom maximum entropy, like Hanoi and Modena (the entropy deviation ∆S is equal to 0.0032 and 0.0616, respectively), and networks with high deviation of entropy, like Parete and BWSN2008-1(entropy deviations of 0.297 and 0.292, respectively).

These differences indicate that the adopted set is suitable to analyze the entropy metrics from a topological point of view in a general sense. 

**Figure 1:**
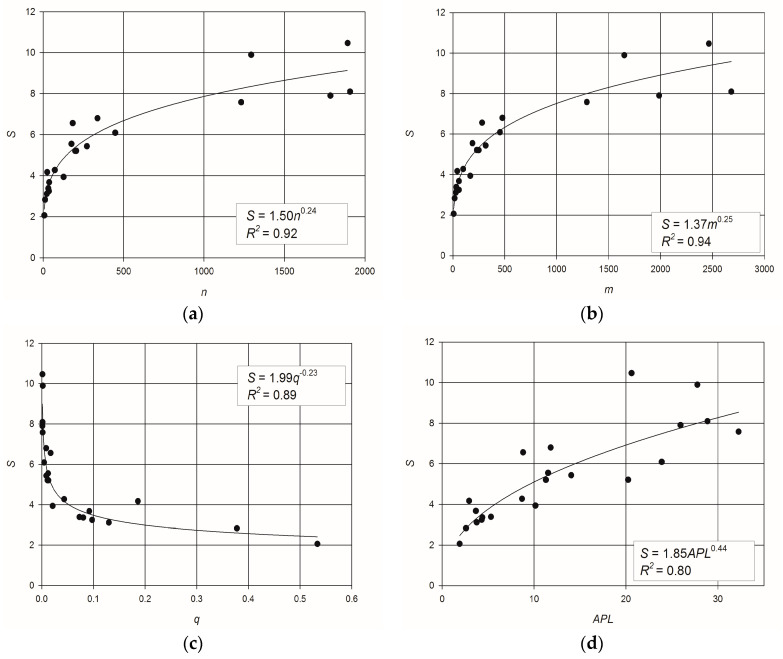
Scatter plots and best fitting power–laws of: (**a**) entropy vs. number of nodes; (**b**) entropy vs. number of pipes; (**c**) entropy vs. link density; (**d**) entropy vs. network average path length.

Figure 1 shows the scatter plots of the values of *S* vs. various topological metrics, and the best fitting power–law equations. The diagrams show that an increasing trend exists in the relationship between flow entropy and number of nodes (Figure 1a), and between flow entropy and number of links (Figure 1b), as well as a decreasing trend for the relationship between flow entropy and link density (Figure 1c). Although in Figure 1d a positive trend of flow entropy vs. average path length is also observable, it is less clearly defined than the previous ones.

**Figure 2:**
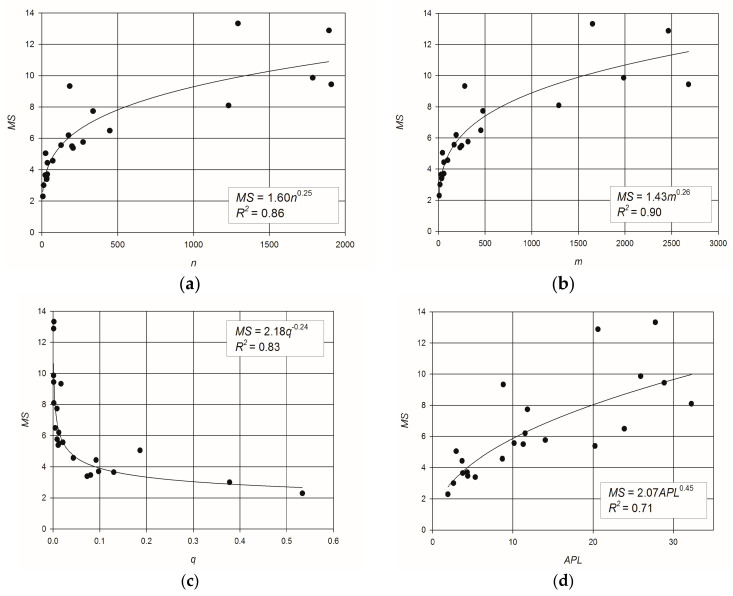
Scatter plots and best fitting power–laws of: (**a**) maximum entropy vs. number of nodes; (**b**) maximum entropy vs. number of pipes; (**c**) maximum entropy vs. link density; (**d**) maximum entropy vs. network average path length.

The scatter plots of *MS* vs. the same topological metrics, reported in Figure 2, confirm similar trends as in Figure 1. Specifically, a clear increasing relation of *MS* with the number of links (Figure 2b) can be noted, while Figure 2d shows a weak relation between *MS* and *APL*. The determination coefficients, *R*^2^, of *S* (Figure 1) are slightly greater than the ones computed for the *MS* (Figure 2). Anyway, both *S* and *MS* are clearly related to network topology.

It is worth to noting that, although *APL* is considered a proxy of the topological robustness of a network [32] and the entropy has been proposed as a surrogate of network reliability, the relationships between *S* and *APL*, as well as between *MS* and *APL*, are less consistent than expected.

The best fitting relationships are the power–laws linking *S* and *MS* with the number of pipes *m*, as indicated by *R*^2^ = 0.94 for the flow entropy and *R*^2^ = 0.90 for the maximum flow entropy (Figure 1b and Figure 2b, respectively). The clear dependence of *MS* and *S* on *m*, indicating that flow entropy is related to the size of the network, suggested to investigate the ratios *S*/*m* and *MS*/*m* to characterize the redundancy of a network regardless of its dimension.

**Figure 3:**
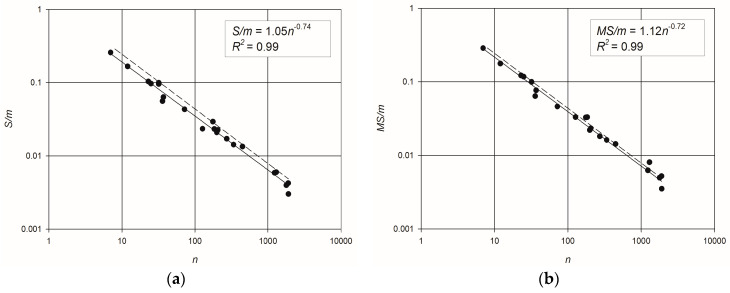
Scatter plots and best-fitting power law equations: (**a**) *S*/*m* vs. number of nodes; (**b**) *MS*/*m* vs. number of nodes. The dashed lines represent the expected scaling of flow entropy for a network with equiprobable flow paths.

The scatter plots of *S*/*m* vs. *n* and *MS*/*m* vs. *n*, and the coefficients of determination of the relevant best fitting power–laws, are reported in Figure 3. A distinct trend is clearly visible for both the flow entropy measures, as indicated by *R*^2^ = 0.99 for both the relationships. The obtained best fitting power–law equations are:

(7) $$S=1.05m\times n^{-0.74}$$

(8) $$MS=1.12m\times n^{-0.72}$$

Looking at Equation (4), and keeping in mind the adopted definition of the probability of the water flowing along a path from a source node to a demand node, it becomes clear that the maximum theoretical flow entropy (i.e., all flow paths sharing the same probability) should scale with lnn. In fact, the number of possible paths in a network scales with the number of nodes (e.g., in a network with a single source, the total number of flow paths to all nodes equals the number of links m=n+l−1, l being the number of loops). Therefore, it is expected that

(9) $$\frac{MS}{m}\sim \frac{-\sum_{n}\frac{1}{n}\ln\frac{1}{n}}{n}=\frac{\ln n}{n}$$

The curve of Equation (9), also plotted in Figure 3, is not far from the scaling behavior exhibited by the maximum entropy of the considered WDNs. The observed difference can be ascribed to the fact that water flows must obey the flow balance equations at nodes, so that equal probabilities of all the flow paths are not physically possible.

It looks clear how both actual and maximum flow entropy strictly depend on network size and topology. The very good alignment of the values of *S* of WDNs designed with different criteria along a single power–law can be seen as an indirect confirmation of its suitability as a measure of network robustness. In fact, regardless of the criteria adopted for the design of pipe diameters, the smaller the hydraulic resistance of pipes (i.e., larger diameter and shorter length), the higher the flows that spontaneously tend to develop through them. The flow distribution along pipes, and so the flow entropy of the network, is thus determined by the hydraulic laws governing energy dissipation along pipes, which lead to the delivery of the demand at nodes with the minimum dissipated power [41] and, at the same time, set limits to the “disorder” of flow distribution.

The small scatter of the points from the curve of Equation (8), comparable to that of Equation (7), is likely due to the imperfect calculation of *MS*, as already discussed in the previous section, due to the a priori assumption of flow directions along pipes. However, as expected, the obtained trend seems not to be significantly affected by such an issue.

**Figure 4:**
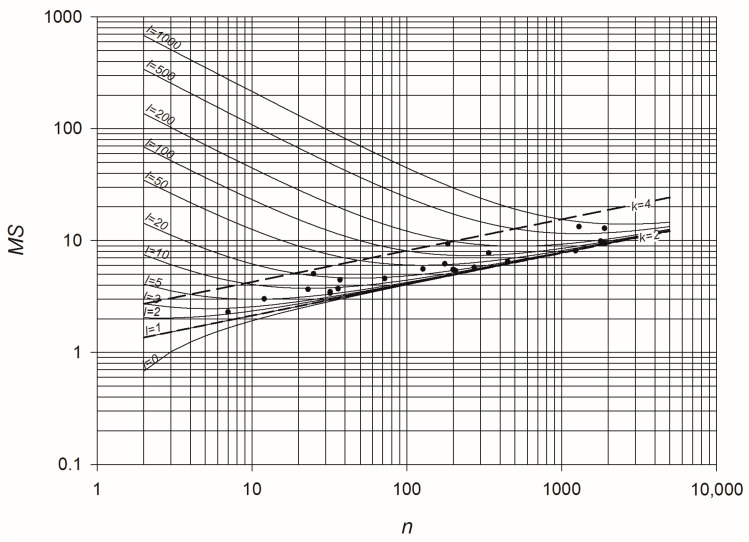
Scaling of maximum flow entropy with number of nodes, for networks with various numbers of loops *l*. The dashed lines represent maximum flow entropy of networks with fixed average node degree k=2 and k=4. The dots represent the considered set of 22 WDNs.

Equations (7) and (8) shed some light on the link between flow entropy and topology of a WDN. In fact, introducing the relationship m=n+l−1, it is possible to compare the flow entropy of networks with different size and different number of loops. In example, Figure 4 shows the dependence of MS on n and l according to Equation (8). It looks clear that the more looped the network is, the higher is its entropy, thus confirming that flow entropy is a suitable measure of WDN redundancy. On the same graph, the curves representing the maximum flow entropy of WDNs with average node degree k=2 and k=4 are also plotted, delimiting the part of the plane to which WDNs belong. In fact, owing to the physical constraints of pipe connections at nodes, the average node degree of most WDNs falls between such values, as confirmed by the positions of the dots representing the 22 considered networks.

The obtained relationships indicate that, thanks to the high values of the coefficients of determination, it is possible to assess the maximum achievable flow entropy of a network starting from mere basic topological information such as the numbers of links and nodes.

In particular, Equation (8) provides a simple way to compute *MS*, without the need of a preliminary determination of flow pipe orientations, which can be easily implemented in the design of water supply networks aiming at taking into account the positive effect of redundancy on network robustness [39].

It is worth highlighting that the obtained relationships (7) and (8) have been derived for very different WDNs, both real and synthetic, from different countries, with quite different topological and hydraulic characteristics. Nonetheless, they show a clear scaling behavior in the form of power–laws, indicating that the values of the informational flow entropy are strongly related to some intrinsic and scale-invariant topological characteristic of WDNs, which likely reflects the spatial embedding of these networks, limiting their topological “disorder” (e.g., the degree connectivity of WDNs assumes a nearly constant value as the size of the network increases [32]). 

## 4. Examples of Application

In this section, practical examples are given of how the maximum flow entropy value *MS*, computed by Equation (8), can be used for WDN design or rehabilitation. Starting from the value of maximum entropy, estimated only by means of topological information, the design of the water supply network can be carried out by means of a multi-objective optimization procedure, based on the minimization of entropy deviation and pipe costs, in compliance with hydraulic constraints (i.e., the required minimum pressure at demand nodes). Specifically, the optimization problem consists of defining the optimal choice of the diameters of all pipes in the network, by minimizing the following multi-objective function (*MOF*):

(10) $$\{\begin{array}{c} MOF=\left\{∆S;\;C=C\prime \cdot \overset{m}{\underset{j=1}{\sum }}L_{j}D_{j}^{\beta }\right\}\\ constraint:h_{i}>\bar{h_{i}}\;i=1,‥,n \end{array}$$

In Equation (10), the first component of *MOF*, ∆*S*, represents the deviation of flow entropy, calculated with Equation (5), from the maximum flow entropy, estimated by means of Equation (8) as a function of *n* and *m*; the second component C represents the total cost of the pipes of the network (C′ is the unit cost of pipes; *L_j_* and *D_j_* are the length and the diameter of the *j*-th pipe, respectively; *β* is a coefficient expressing the dependence of the cost of a pipe on its diameter, for which the value β=1.5 has been proposed [69]); $h_{i}$ and $\bar{h_{i}}$ are, respectively, the actual and the design pressure heights at the *i*-th node of the network.

**Figure 5:**
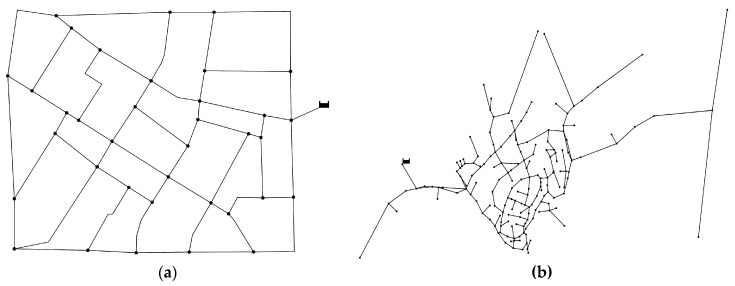
Layouts of the water distribution networks for which the multi-objective optimal design procedure based on maximum flow entropy has been applied: (**a**) Fossolo; (**b**) Skiathos.

The application of the proposed WDN design optimization procedure, summarized by Equation (10) , has been carried out for the real water supply networks of Fossolo [60], a neighborhood of the city of Bologna (Italy), and of the town of Skiathos (Greece) [62]. The first network consists of 36 nodes and 58 polyethylene pipes, and the design pressure was assumed equal to $\bar{h}$ = 30 m at all nodes. The second network, made with cast iron pipes, has n=175 and m=189, with $\bar{h}=22$ m. In Figure 5, the sketches of the WDN of Fossolo and Skiathos are reported.

The minimization of *MOF* was carried out by a heuristic optimization method based on a Genetic Algorithm (GA), a minimum search technique based on mimicking the process of natural selection in the evolution of species [70]. Such an evolutionary algorithm allows for easily introducing constraints on the unknown parameters, at the same time avoiding local minima by introducing random variations to parameter vectors. The GA parameters are the following: each individual of the population is a sequence of chromosomes corresponding to the diameters of all the pipes of the network, which can assume only the values of the existing commercial pipes reported in Table 2. The number of GA generations, the size of the population and the crossover percentage were set to 100, 100 individuals and 0.8, respectively.

**Figure 6:**
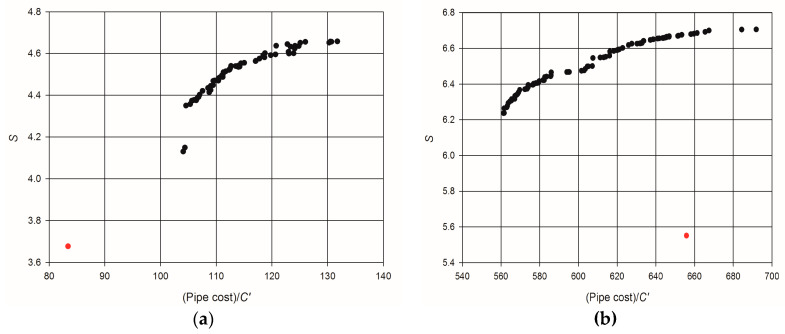
Pareto fronts of the proposed multi-objective optimal network design procedure (flow entropy and total cost of network pipes): (**a**) Fossolo; (**b**) Skiathos. The red dots correspond to network layouts before optimization.

The application of the proposed network design procedure led to the definition of the Pareto frontsreported in Figure 6, which represent, in the plane (C,S), the set of all the optimal solutions obtained by minimizing ∆*S* and the total pipe cost, in compliance with the hydraulic constraints of Equation (10). In addition, the red dots in Figure 6 represent the entropy deviation and the total pipe cost of the original network layouts. Without limiting the general validity of the obtained results, the unit cost of pipes has been assumed C′=1. The obtained Pareto fronts show that the smallest values of ∆*S* correspond to the highest values of total pipe cost, as the more a network is robust, the more investment is needed for its realization (e.g., [71]). For the network of Fossolo, the minimum value of ∆*S* = 0.0004, corresponding to a flow entropy S=4.66, implies an increase of pipe cost, compared with the original layout, of about 58%. However, a flow entropy S=4.55 can be obtained with an increase of cost smaller than 25%, which represents a good tradeoff between reliability improvement and cost increase. For the case of Skiathos, instead, it is worth noting that nearly the maximum flow entropy S=6.72 can be achieved without any increment of overall pipe cost compared to the existing network.

## 5. Conclusions

The study investigates the scaling-law of informational flow entropy of water distribution networks, often assumed as a surrogate of network robustness, with their topological size. To such aim, the relationships between informational flow entropy, *S*, maximum informational flow entropy, *MS*, and some suitable topological metrics (namely, number of nodes, *n*; number of links, *m*; network link density, *q*; network average path length, *APL*) are investigated for a set of 22 networks, both real and synthetic, with different characteristics. 

A clear dependence of flow entropy on topological metrics is observed, and, in particular, power–law relationships, strongly linking *S*/*m* and *MS*/*m* to the number of nodes of the network (i.e., *R*^2^ = 0.99), are identified. The obtained scaling laws result in being close to the expected scaling of flow entropy in networks with equiprobable flow paths (i.e., the same flow carried to the end of any flow path connecting sources to demand nodes), although the actual flow paths cannot be equiprobable, as they must obey flow balance equations at nodes. Such a scale-invariant behavior, testified by the power–laws, probably reflects the peculiar topological feature of water distribution networks, in which each node is connected only to a few immediately surrounding nodes, thus limiting the topological “disorder” of the network, i.e., the number of possible flow paths from each node.

The obtained power–laws, providing an easy estimate of actual and maximum flow entropy of a network, allow to quantify the entropy deficit of a network, i.e., the distance of the flow entropy of a network of given topology from its maximum achievable flow entropy, which can be used in network design and rehabilitation as a measure of network robustness. In this respect, examples of application to multi-objective design of real water distribution networks show how optimal solutions in terms of pipe cost and overall network robustness are easily identified.

## Figures and Tables

**Table 1:** Topological metrics: number of nodes (*n*) and links (*m*), density (*q*), average path length (*APL*), flow entropy (*S*) and maximum flow entropy (*MS*), for all WDN (* denotes synthetic networks).

Network	*n*	*m*	*q*	*APL*	*S*	*MS*	∆S
Two Loop * [53]	7	8	0.5333	1.90	2.063	2.296	0.101
Two Reservoirs * [54]	12	17	0.3778	2.59	2.829	3.008	0.059
Anytown * [55]	25	43	0.1861	2.94	4.172	5.048	0.174
GoYang * [56]	23	30	0.1299	3.75	3.113	3.658	0.149
Blacksburg * [57]	32	35	0.0805	4.37	3.358	3.473	0.033
Hanoi * [58]	32	34	0.0731	5.31	3.384	3.395	0.003
BakRyan * [59]	36	58	0.0975	4.30	3.243	3.709	0.126
Fossolo [60]	37	58	0.0921	3.67	3.677	4.441	0.172
Pescara [60]	72	99	0.0435	8.69	4.273	4.572	0.065
BWSN2008-1 * [61]	127	168	0.0213	10.15	3.939	5.567	0.292
Skiathos [62]	176	189	0.0124	11.52	5.551	6.196	0.104
Parete [1]	184	282	0.0171	8.80	6.561	9.331	0.297
Villaricca [1]	199	249	0.0130	11.29	5.206	5.497	0.053
Monteruscello [63]	206	231	0.0110	20.24	5.211	5.385	0.032
Modena [60]	272	317	0.0089	14.04	5.436	5.764	0.057
Celaya [64]	338	477	0.0086	11.81	6.8	7.734	0.121
Balerma Irrigation [65]	448	454	0.0046	23.89	6.091	6.489	0.061
Castellammare	1231	1290	0.0017	32.25	7.583	8.094	0.063
Matamoros [66]	1293	1651	0.0020	27.76	9.896	13.325	0.257
Wolf Cordera Ranch [67]	1786	1985	0.0013	25.94	7.905	9.865	0.199
Exnet * [68]	1893	2465	0.0014	20.60	10.466	12.882	0.188
San Luis Rio Colorado [66]	1908	2681	0.0015	28.86	8.097	9.443	0.143

**Table 2:** Pipe diameters of the networks of Fossolo (polyethylene pipes) and Skiathos (cast iron pipes).

FossoloDN (mm)		SkiathosDN (mm)	
16.00	73.60	40.00	125.00
20.40	90.00	50.00	140.00
26.00	102.20	63.00	150.00
32.60	147.20	75.00	160.00
40.80	184.00	80.00	225.00
51.40	204.6	90.00	
61.40	229.2	110.00	
